# Effects of Annealing on the Residual Stress in γ-TiAl Alloy by Molecular Dynamics Simulation

**DOI:** 10.3390/ma11061025

**Published:** 2018-06-15

**Authors:** Ruicheng Feng, Wenyuan Song, Haiyan Li, Yongnian Qi, Haiyang Qiao, Longlong Li

**Affiliations:** 1 School of Mechanical and Electronical Engineering, Lanzhou University of Technology, Lanzhou 730050, China; frcly@163.com (R.F.); 18109423072@163.com (W.S.); 13893591492@163.com (Y.Q.); qiaohaiyanglut@163.com (H.Q.); 18194210274@163.com (L.L.); 2 Key Laboratory of Digital Manufacturing Technology and Application, Ministry of Education, Lanzhou University of Technology, Lanzhou 730050, China

**Keywords:** residual stress, molecular dynamics, γ-TiAl alloy, anneal

## Abstract

In this paper, molecular dynamics simulations are performed to study the annealing process of γ-TiAl alloy with different parameters after introducing residual stress into prepressing. By mainly focusing on the dynamic evolution process of microdefects during annealing and the distribution of residual stress, the relationship between microstructure and residual stress is investigated. The results show that there is no phase transition during annealing, but atom distortion occurs with the change of temperature, and the average grain size slightly increases after annealing. There are some atom clusters in the grains, with a few point defects, and the point defect concentration increases with the rise in temperature, and vice versa; the higher the annealing temperature, the fewer the point defects in the grain after annealing. Due to the grain boundary volume shrinkage and and an increase in the plastic deformation of the grain boundaries during cooling, stress is released, and the average residual stress along Y and Z directions after annealing is less than the average residual stress after prepressing.

## 1. Introduction

Due to its low density, high specific strength, excellent high-temperature properties, good oxidation resistance and creep resistance, γ-TiAl alloy is considered to be the high-temperature structural material with the most potential. It has wide application prospects in many fields, such as aerospace and so on [1,2,3,4]. However, poor ductility at room temperature greatly restricts the application of γ-TiAl alloy, and introduces residual stress during its forming and processing; the main problems of γ-TiAl alloy are surface deformation, higher surface roughness and residual stress, defects that will become initial crack-extension points, leading to workpiece failure [5,6]. Residual stress is also the root cause of workpiece deformation during the manufacture–service process. The magnitude and distribution of residual stresses have played a major role in the stability of the workpiece, which directly affects its service life and mechanical properties [7].

Lots of work has been done to obtain the ideal residual stress distribution and improve the service life of the workpiece, especially on the relationship among process parameters and the generation mechanism of residual stress and the method of eliminating that stress. Pawade et al. [8] proved the effects of cutting speed, cutting depth, feed rate and tool geometry parameters on residual stress by an experimental method; a residual compressive stress could be produced on a machined surface by adjusting cutting parameters and tool shape properly. Cheng et al. [9] combined the cutting experiment results with finite element analysis methods, and found that the combination of cutting force and cutting heat produced high temperature and high pressure when acting on the rake face of the cutting tool, and inhomogeneous plastic deformation were introduced which led to residual stress. The effect of cutting force and temperature on residual stress in the milling process has been investigated by Jiang et al. [10], and the results show that the cutting force plays a leading role in the residual stress, and the tangential residual stress was mainly caused by the tangential force and temperature. Szczepan et al. [11] has studied the residual stresses in a hot-rolled steel strip during cooling in coils, and found that the phase transformations have a significant influence on the level of residual stress. Xiao et al. [12] analyzed the thermal residual stress in glass/glass laser bonding, and the results show that the scale of the temperature field control is closely related to the residual stress, and that by combining the point, line and surface-scaled control, the thermal residual stress can be reduced. A new multistage aging method has been exploited in reducing residual stresses in the quenching process by Sun et al. [13]. Laser shock peening of austenitic stainless steel has been researched by Prabhakaran et al. [14], who found that the residual compressive stress of the material can be increased by adjusting the pulse parameters. R Sola et al. [15,16] used the experimental methods to study the application of the cryogenic treatment and post-tempering cryogenic treatment of AISI M2, AISI D2, X105CrCoMo18 steels. The results show that the precipitation of carbides that occurs during heating from the cryogenic treatment temperature is responsible for the residual stress relaxation, and the precipitation of more hard carbides in the cryogenically treated samples can reduce residual stresses and also enhance the steel fracture toughness. Shao H et al. [17] has investigated joule-induced microstructure evolution and residual stress in a Ti–Al–4V U-shaped screw by an experimental method, and the results show that dislocation density decreases with increasing heated time; joule heating at 900 °C is sufficiently high to enhance the dislocation mobility and the rearrangement, causing the recrystallization of the alpha phase and the change of residual stress. The effect of annealing on the microstructure and residual stress of zirconium has been researched by Zhang C H et al. [18], who found that grain size increases after annealing, and that the decrease of dislocation density and the rearrangement of dislocation after annealing are closely related to the release of residual stress. 

Through a large number of experiments, it has been found that the generation and elimination of residual stress in a workpiece, and the mechanical properties of workpiece material, are closely related to the microstructure of the material. Yang et al. [19] puts forward the concept of “making materials plain” that considers that the material properties can be improved by regulating the defects of materials at different scales. Wawszczak et al. [20] has studied the evolution of microstructure and residual stress during annealing at different temperatures. The results show that the stress in the samples would be relaxed subjected to heat treatment, and the stress in the samples was correlated with the progress of recovery process which depends on the value of stacking fault energy. Marzbanrad et al. [21] has found grain refinement on the substrate surface results in higher residual compressive stress during a cold spray process. The evolution of microstructure and residual stress during rapid thermal annealing is observed by Hsiao et al. [22]. It is considered that the tensile stress of the film originates from the annihilation of L1_0_ grain boundaries in single-layered FePt films. Zhao et al. [23], has found that the lattice strain caused by thermal expansion mismatch between perovskite and substrate is an important factor affecting the stability of perovskite solar cells. This residual strain is caused by the annealing process in the preparation of perovskite thin films. Nakano et al. [24] has explained the relationship between dislocation density and residual stress in a GaN single crystal during the cooling process by numerical analysis, with the results showing that the residual stress increases with the rise of dislocation density during cooling.

As a powerful supplement to the experiment and the verification of theoretical model, molecular dynamics (MD) methods are used to simulate the relationship among microdefects’ evolution, processing parameters and the mechanical properties of the processed materials [25,26,27,28]. Although ample research has been conducted on the evolution of microstructure and residual stress, there is little research on the distribution of residual stress in annealing of γ-TiAl alloys. In this paper, we seek to better understand: the effects of annealing on residual stress and improve the annealing process of γ-TiAl alloy; and investigate the distribution of residual stress after annealing, the microdefects evolution during annealing, and the relationship between microstructure and residual stress with MD simulations. In Section 2, the simulation model and details will be introduced. In Section 3, the simulation results will be obtained and the corresponding analysis undertaken. Finally, some conclusions will be drawn in Section 4.

## 2. Simulation Details

### 2.1. Interatomic Potential

The effects of vacancy concentration and temperature on mechanical properties of single-crystal γ-TiAl have been carried out by using the Large Scale Atomic/Molecular Massively Parallel Simulator (Sandia National Laboratories, Albuquerque, NM, USA) (LAMMPS) [29]. It is widely believed that interatomic potentials are important for MD simulations, and the selection of them critically affects the accuracy of the MD simulation. For example, the embedded atom method (EAM) has been used to study phase transformation of Ti–Al alloy and the defects and their evolution on crack propagation behavior [30,31]. In this paper, EAM is employed to describe the interaction of atoms between materials. 

### 2.2. Molecular Dynamics (MD) Model

**Figure 1:**
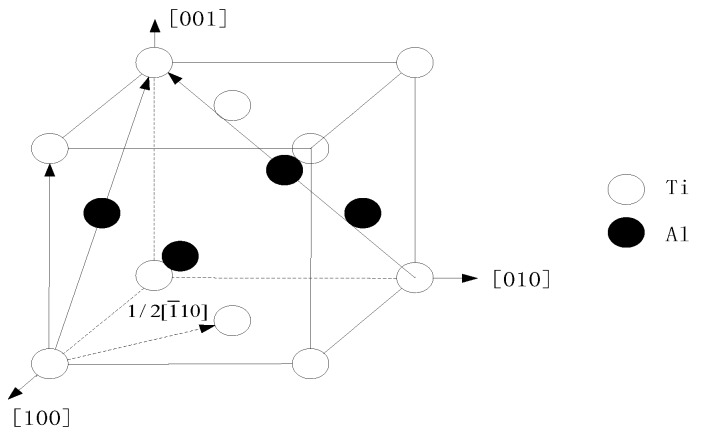
The crystal structure of γ-TiAl alloy.

The crystal structure of γ-TiAl alloy is L1_0_ [32,33] which is shown in Figure 1; the lattice constants are a = 4.001, b = 4.001, c = 4.181, respectively. 

**Figure 2:**
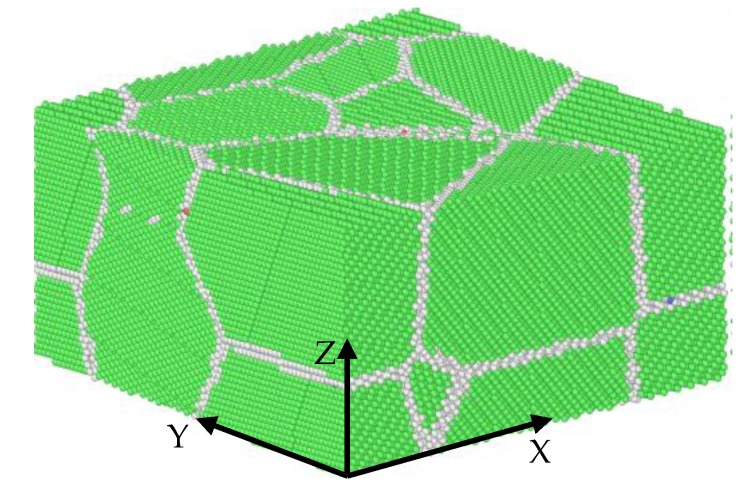
The simulation model.

The initial model is created using the ATOMSK package by means of a Voronoi tesselation to construct polycrystals, which is shown in Figure 2, where the green area is the grain and the white area is the grain boundary. 

The model contains 239,084 atoms and has a size of 200a × 200b × 100c which contains eight unit cells with different random directions; all three directions are periodic boundary conditions. 

### 2.3. Definition of Residual Stress 

The stress at any point in the model is completely defined by nine stresses, and can be expressed as a second-order tensor as follows [34]:

(1) $$\sigma_{ij}=\left(\begin{array}{ccc} \sigma_{11} & \sigma_{12} & \sigma_{13}\\ \sigma_{21} & \sigma_{22} & \sigma_{23}\\ \sigma_{31} & \sigma_{32} & \sigma_{33} \end{array}\right),$$

**Figure 3:**
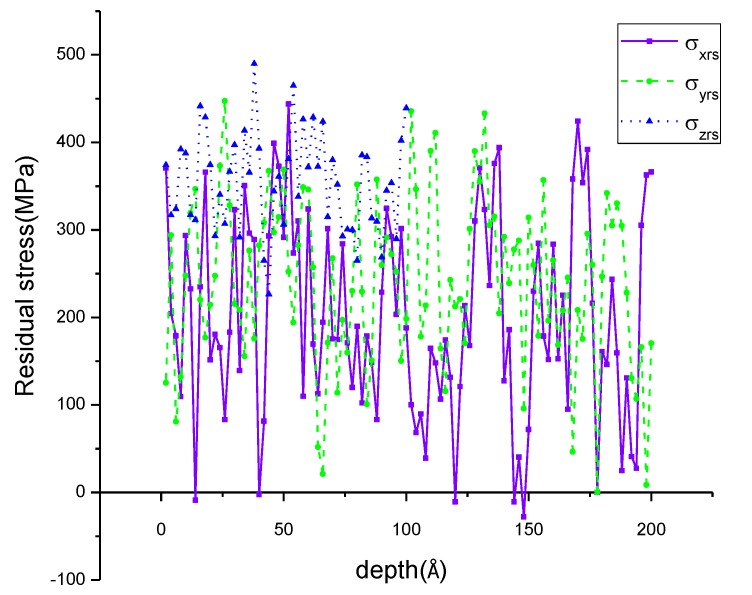
The residual stress in the X, Y, Z directions after prepressing.

In this paper, the stress after fully relaxing is defined as residual stress which is calculated by virial theory [35]. Moreover, the residual stress is obtained for each atom by averaging the output data of the standard LAMMPS command “compute stress/atom” over the region within a range of 2 Å along the corresponding directions. Define *σ_xrs_*, *σ_yrs_*, *σ_zrs_* are the residual stress in the X, Y, Z directions. The residual tensile stress was introduced into the model by a 2% prepressing deformation in the Z direction before annealing. The distribution of *σ_xrs_*, *σ_yrs_*, *σ_zrs_* after prepressing is shown in Figure 3. The maximum residual tensile stress in the X, Y, Z directions is 443.75 MPa, 447.25 MPa, 489.875 MPa, respectively, and the corresponding depths are 54 Å, 28 Å, 40 Å.

The MD using a constant-pressure, constant-temperature ensemble (NPT ensemble), firstly relaxes at 30 ps, and the annealing simulation is carried out after reaching equilibrium, using the Nose–Hoover thermostat for the temperature control. The annealing processes are divided into four cases, and the annealing parameters are shown in Table 1. The timestep is 0.001 ps; kinetic energy, potential energy and total energy are recorded every 500 steps during annealing.

## 3. Results

### 3.1. Structural Evolution in Annealing

**Figure 4:**
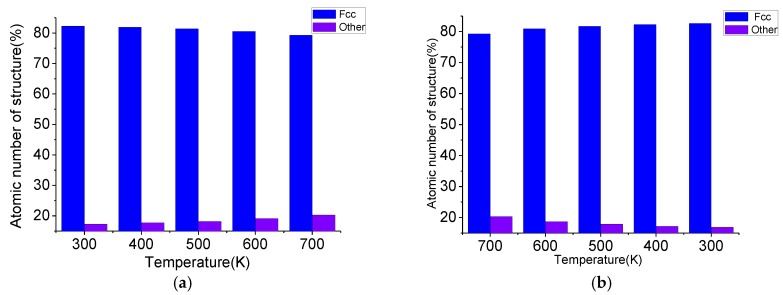
The atomic number of the structure during annealing: (**a**) the atomic number of the structure during heating; (**b**) the atomic number of the structure during cooling.

**Figure 5:**
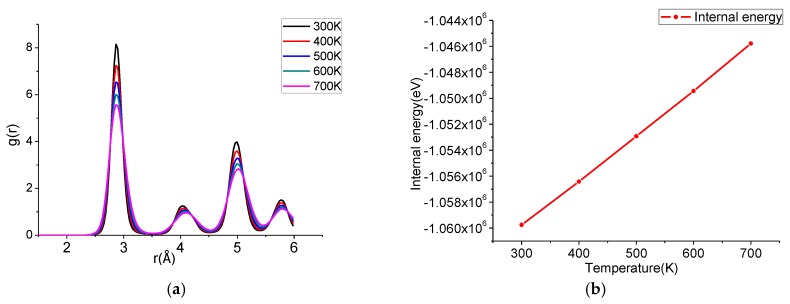
The radial distribution functions (RDF) curve during annealing: (**a**) the RDF curve during heating; (**b**) the internal energy curve during heating.

Taking Case 1 as an example, the atomic number of the structure during annealing is shown in Figure 4. In this paper, common neighbor analysis (CNA) is employed to analyze the atoms’ distortion. The γ-TiAl alloy atoms with L1_0_-type structure belong to the recognizable FCC lattice structure, while the lattice structures corresponding to the atoms at the grain boundary are atypical other lattice structures. In order to avoid the interference of surface atoms, the surface atoms were selected and then eliminated by centrosymmetric parameters. The number of FCC atoms decreases gradually while the other type atoms increase during heating, which means that the grain boundary volume expands when the temperature rises, and the grains are compressed. By observing the output of the internal energy diagram and the radial distribution functions (RDF) curve as shown in Figure 5, it can found that the internal energies persistently and smoothly increase with the rise of temperature. The RDF curves illustrate that the structure is a classic crystal state shape at elevated temperature, there are four independent peaks, and the value of curves between peaks is zero, which indicates that the lattice structure of the grain has good long-term order. There is a certain phenomenon of broadening of the peaks during heating, which means that the crystal atoms are not exactly in the ideal lattice position, and the lattice order is reduced. The FCC atoms increase and the other atoms decrease when cooling; also, the internal energies persistently decrease with the drop of temperature, the RDF curve peak width decreases gradually, and the lattice order increases gradually when the temperature drops. After cooling down to 300 K and fully relaxing, the average grain size slightly increased from 4.639 nm to 4.643 nm. In combination with the variation of internal energy, the RDF curve and the atomic number of the structure, it is found that there is no phase transition during annealing, but the atoms’ distortion occurs with the change of temperature. 

### 3.2. Microdefects Evolution during the Annealing

**Figure 6:**
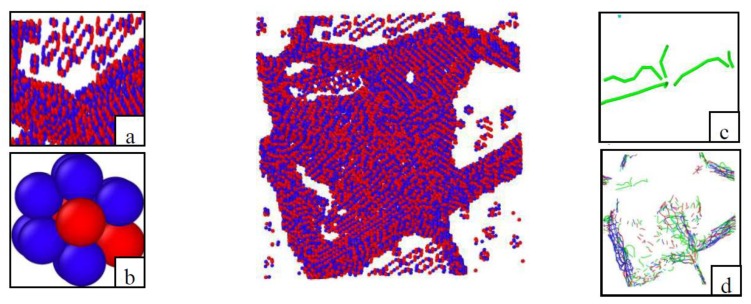
Microdefects in the initial model after prepressing: (**a**) the atom clusters in the clusters’ grains; (**b**) a Ti anti-site point defect (blue atoms are the Ti atoms, red atoms are the Al atoms); (**c**) the dislocations at the atom clusters; (**d**) the dislocations at the grain boundaries.

To observe the evolution of the microdefects, their distribution after prepressing is shown in Figure 6, with use of the CNA analysis to identify the defect atoms and then delete the non-defective normal atoms. After prepressing plastic deformation, there are some atom clusters in the crystal grains, some atom clusters are neatly arranged in a ring, some atom clusters are gathered together disorderly with few dislocations, and there are a few point defects in the crystal grains. There are different types of dislocations at the grain boundaries after prepressing. 

**Figure 7:**
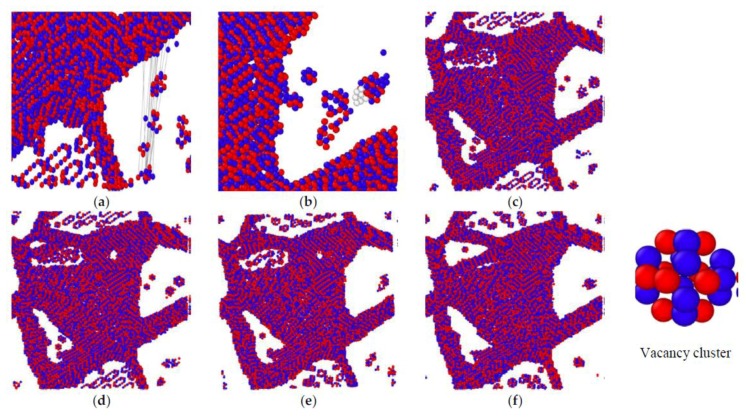
The distribution of the point defect after annealing. (**a**) the precipitate atoms from which from grain boundaries comes when temperature rises, and the gray lines are the atomic trajectory; (**b**) the white atoms adsorb on the atom clusters which are precipitated from the grain boundaries; (**c**–**f**) is the distribution of the point defects in the grain after annealing in Case 1, Case 2, Case 3, Case 4.

The change of temperature has a great influence on the point defect concentration of Ti–Al alloy [36]. In general the energy of the point defect formation is based on the Arrhenius equation which can be given as follows:

(2) $$C=Aexp\left(-\frac{Q}{RT}\right),$$

where C is point defect concentration, A is the equilibrium constant, Q is the point defect formation activation energy, R is the molar constant, and T is the temperature. According to Equation (2), it can be derived that the point defect concentration increases with the rise of temperature. The variation trend of the point defect concentration in the simulation results is consistent with the theoretical calculation. We found that the grain boundaries precipitate atoms to form the atom clusters when the temperature rises by tracing the atomic trajectory, as shown in Figure 7a. The atom which precipitated from the grain boundaries firstly tends to move to the atom cluster (Figure 7b); this phenomenon is similar to [37]. It was found that small vacancy clusters have the ability to capture vacancies. The atom clusters decompose during cooling and the atoms enter the grain boundaries. Figure 7c–f represents the distribution of point defects of Case 1, Case 2, Case 3, Case 4 after annealing, respectively. It can be seen that the higher the annealing temperature, the fewer the point defects in the grain after annealing. Due to insufficient cooling time, after annealing at 1100 K a vacancy cluster still exists in the grain which cannot decompose completely, and the difference in the distribution of point defects between Case 1 and Case 2 after annealing is very small. According to [36,37], the enthalpy of formation and the formation energy of the anti-site defect is less than that of the vacancy defect, suggesting the anti-site defect can be seen in the grain after annealing.

### 3.3. Residual Stress Distribution after Annealing with Different Parameters

**Figure 8:**
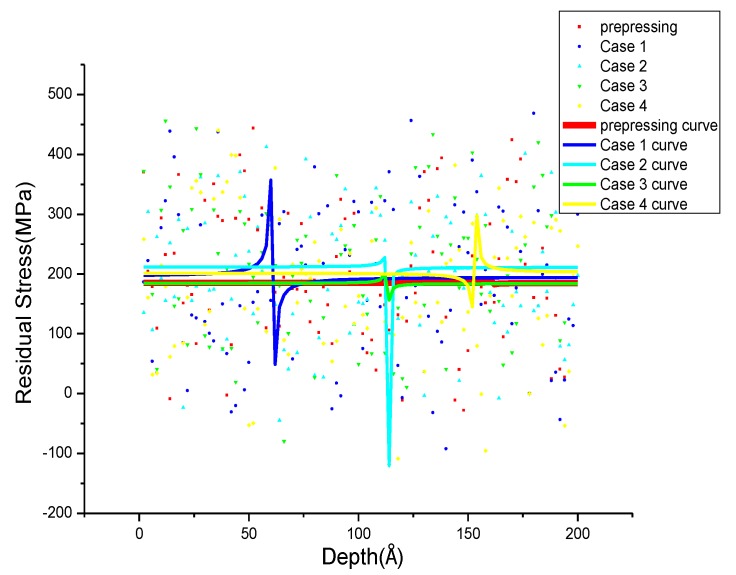
*σ_xrs_* distribution after annealing with different annealing parameters.

**Figure 9:**
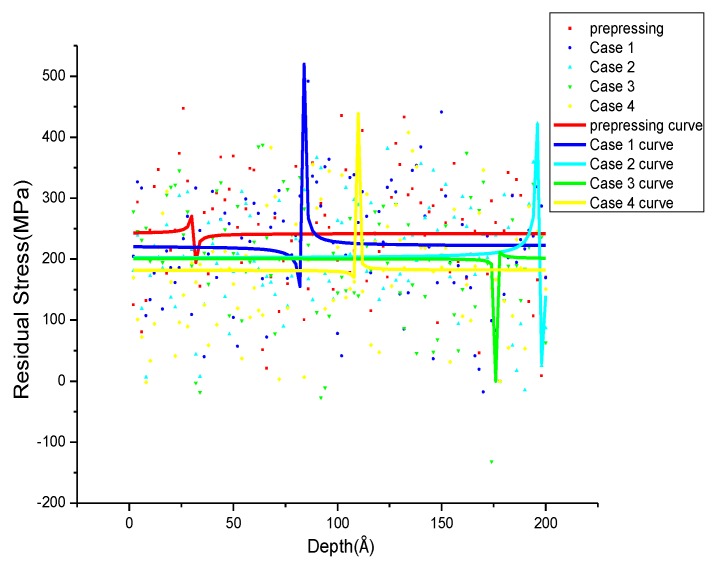
*σ_yrs_* distribution after annealing with different annealing parameters.

**Figure 10:**
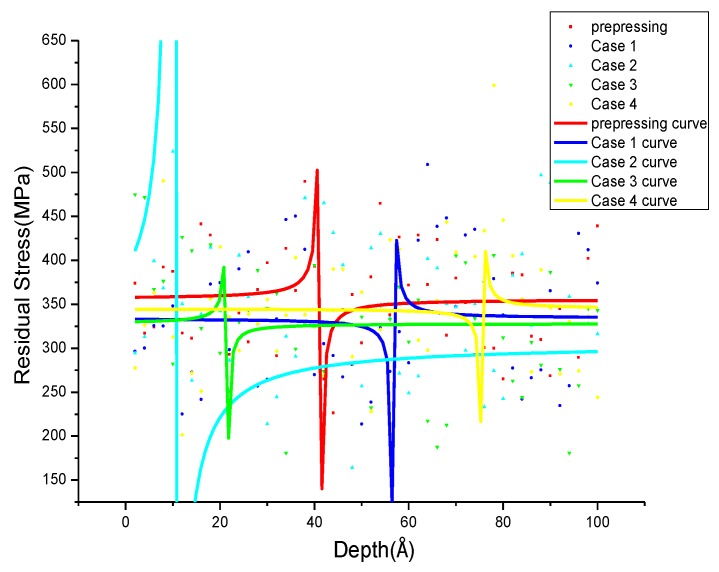
*σ_zrs_* distribution after annealing with different annealing parameters.

The distribution of *σ_xrs_*, *σ_yrs_*, *σ_zrs_* after annealing with different parameters is shown in Figure 8, Figure 9 and Figure 10. To observe the distribution of residual stress in three directions more intuitively, non-linear fitting of the data is carried out by using rational functions. In Figure 8, the fitting average value of the residual stress along the X-direction after annealing is 184 to 212 MPa, and the *σ_xrs_* curve after prepressing almost overlapped with Case 3. The distribution of *σ_xrs_* with different annealing parameters in the specific position is different, and the residual stress fluctuation is small in other positions. The difference in residual stress distribution is larger at 50–72 Å in Case 1, and the *σ_xrs_* shows a sharp drop in Case 2 at 110 Å to 116 Å. In Case 3 and Case 4, the residual stress decreases first and then increases at 112–116 Å and 150–160 Å. In Figure 9, the average residual stress along the Y direction after annealing in all four cases is less than the average residual stress after prepressing. The higher the annealing temperature, the smaller the average residual stress and at the same annealing temperature it can obtain smaller average residual stress after annealing at a slower cooling rate. The *σ_yrs_* increases sharply at 82 Å to 90 Å, 108–112 Å in Case 1 and Case 4, respectively. The residual stress of Case 3 decreases in the range of 174 Å to 178 Å; the difference of *σ_yrs_* distribution in Case 2 before 184 Å is small, and it increases first and then decreases from the range of 184 Å to 200 Å. In the Z direction, the average residual stress after annealing is almost lower than the average residual stress after prepressing. In Case 1 and Case 4, the residual stress decreases first and then increases at 52 Å to 60 Å and 72 Å to 80 Å. The *σ_zrs_* in Case 3 increases first and then decreases at 12–26 Å. In Case 2, there are large difference in *σ_zrs_* before 14 Å and these then tend to 300 MPa which is lower than the average residual stress after prepressing. The smaller the cooling rate at the same annealing temperature, the lower the average residual stress after annealing.

**Figure 11:**
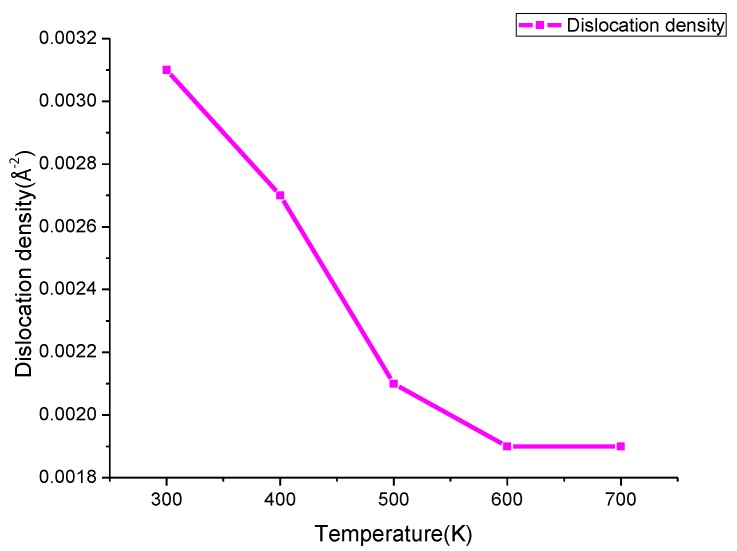
The trend of dislocation density during cooling in Case 1.

From Figure 7c–f, it can be seen that after annealing, the point defect concentration in thermal equilibrium is small, but the residual stress distribution is very different. Therefore, the point defect concentration is not the main factor affecting the residual stress distribution. Hammer et al. [38] investigated the residual stress gradient in W/TiN-stack on Si(100). They found that stress profiles can be related to grain size distribution by a Hall–Petch mechanism: in order to avoid extreme residual stress in metallic films, an increase the grain size can hence be suggested, as this decreases the yield strength and effectively limits the maximum possible stress level. The yield stress is related to the grain size by the Hall–Petch law equation which can be given as follows [39]:

(3) $$\sigma_{y}=\sigma_{0}+\frac{k}{\sqrt{d}},$$

where *σ_y_* is the yield stress, *σ*_0_ is approximately the yield stress of a very coarsegrained, untextured polycrystal, *k* is the strength coefficient, *d* is the average grain size, and *σ*_0_ and *k* are constants. For γ structure, *σ_y_* = 175 + 0.615/*d* MPa [40]. However, an “inverse Hall–Petch” phenomenon occurs when the grain size is less than 10 nm because the plastic deformation of the grain boundaries replaces the dislocation plasticity mechanism within the grain when the grain boundary is excessive [41,42]. The average grain size in four cases after annealing is 4.643 nm, 4.655 nm, 4.658 nm, 4.657 nm, respectively, and more than 4.639 nm after prepressing; the average grain size increases slightly after annealing. In general, according to the “inverse Hall–Petch” phenomenon the yield stress has been increased after annealing and the residual stress may be increased after annealing. However, the fitting average residual stress in Y and Z directions show that the average residual stress after annealing in all four cases is less than the average residual stress after prepressing. The reason for this phenomenon is that the plastic deformation of the grain boundaries influences the distribution of residual stress. The simulation results show that during the annealing process, the grain boundary dislocation density decreases when heating; similar results are found in [17] when joule heating a Ti-Al-4V U-shaped screw. The dislocation increases during cooling with few dislocations in the grain. The decrease of dislocation density after annealing is closely related to the release of residual stress [18]. Table 2 shows the dislocation density after annealing with different parameters and, taking Case 1 as an example, the tend of dislocation density is shown in Figure 11. The grain boundary dislocation density after annealing is slightly larger than that after prepressing. The volume of grains increases and the grain boundary volume shrinks during cooling, the dislocation density at grain boundaries increases gradually which causes the grain boundary plastic deformation to increase, and stress is released. This finally causes the fitting average residual stress in the Y/Z direction after annealing to decrease.

## 4. Conclusions

In this paper, annealing processes of γ-TiAl alloy after introducing residual stress into prepressing are simulated, and the dynamic evolution process of microdefects and the distribution of residual stress before and after annealing are investigated. The conclusions are as follows:

(1) The grain boundary volume expands when the temperature rises, and the grains are compressed. The volume of the grain boundary shrinks when the temperature is dropped, and the grain size slightly increases after annealing. There is no phase transition during annealing, but the atoms’ distortion occurs with the change of temperature.
(2) There are some atom clusters in the grains, with a few point defect and dislocations, and the main defects at the grain boundaries are different types of dislocation after prepressing. The point defect concentration increases with the rise of temperature and vice versa. The atom clusters have a certain adsorption effect on the atoms that precipitated from the grain boundaries. The higher the annealing temperature, the less the point defects in the grain after annealing.
(3) The distribution of residual stress in the X direction fluctuates slightly. In the Y direction, the higher the annealing temperature, the smaller the average residual stress, and the same annealing temperature can obtain smaller average residual stress after annealing at a slower cooling rate. In the Y and Z directions, the average residual stress after annealing in all four cases is less than the average residual stress after prepressing; the reason for this phenomenon is the grain boundary volume shrinkage and plastic deformation of the grain boundaries increases during cooling, and stress is released.

## Figures and Tables

**Table 1:** Annealing parameters.

Case	Annealing Temperature (K)	Heating Rate (K/ps)	Cooling Rate (K/ps)	Holding Temperature (K)
1	700	2	2	700
2	700	2	1	700
3	900	2	2	700, 900
4	1100	2	2	700, 1100

**Table 2:** Dislocation density at grain boundaries after annealing.

Case	Annealing Temperature (K)	Dislocation Density (Å^−2^)
1	700	0.0031
2	700	0.00278
3	900	0.0031
4	1100	0.00281
prepressing	/	0.00248

## References

[1] Bewlay B.P., Weimer M., Kelly T., Suzuki A., Subramanian P.R. (2013). The Science, Technology, and Implementation of TiAl Alloys in Commercial Aircraft Engines. MRS Proc..

[2] Deshuang Z., Ruirun C., Jian W., Tengfei M., Hongsheng D., Yanqing S., Jingjie G., Hengzhi F. (2017). Novel casting TiAl alloy with fine microstructure and excellent performance assisted by ultrasonic melt treatment. Mater. Lett..

[3] Deshuang Z., Ruirun C., Jian W., Tengfei M., Hongsheng D., Yanqing S., Jingjie G., Hengzhi F. (2017). The microstructure, mechanical properties, and oxidation behavior of betagamma TiAl alloy with excellent hot workability. Mater. Sci. Eng. A.

[4] Kim Y.W., Kim S.L. (2018). Advances in Gammalloy Materials–Processes–Application Technology: Successes, Dilemmas, and Future. JOM.

[5] Xu W.F., Yan R., Liu W.W. (2017). Surface residual stress of Gamma Titanium Aluminide in milling process. Aeronaut. Manuf. Technol..

[6] Yao C., Lin J., Wu D., Ren J. (2017). Surface integrity and fatigue behavior when turning γ-TiAl alloy with optimized PVD-coated carbide inserts. Chin. J. Aeronaut..

[7] Ball D. Improved assessment of the impact of bulk residual stress on the life, weight and cost of aircraft structure. Proceedings of the International Conference on Experimental Mechanics.

[8] Pawade R.S., Joshi S.S., Brahmankar P.K. (2008). Effect of machining parameters and cutting edge geometry on surface integrity of high-speed turned Inconel 718. Int. J. Mach. Tools Manuf..

[9] Cheng Y., Xu M., Guan R., Liu L., Qian J. (2017). Generation mechanism of insert residual stress while cutting 508III steel. Int. J. Adv. Manuf. Technol..

[10] Jiang X., Li B., Wang L., Li H. (2016). An approach to evaluate the effect of cutting force and temperature on the residual stress generation during milling. Int. J. Adv. Manuf. Technol..

[11] Szczepan W., Andrij M. (2018). Numerical analysis of temperature and residual stresses in hot-rolled steel strip during cooling in coils. Arch. Civ. Mech. Eng..

[12] Xiao Y., Wang W., Wu X., Zhang J. (2017). Process design based on temperature field control for reducing the thermal residual stress in glass/glass laser bonding. Opt. Laser Technol..

[13] Sun Y., Jiang F., Zhang H., Su J., Yuan W. (2016). Residual stress relief in Al–Zn–Mg–Cu alloy by a new multistage interrupted artificial aging treatment. Mater. Des..

[14] Prabhakaran S., Kulkarni A., Vasanth G., Kalainathan S., Shukla P., Vasudevan V.K. (2018). Laser shock peening without coating induced residual stress distribution, wettability characteristics and enhanced pitting corrosion resistance of austenitic stainless steel. Appl. Surf. Sci..

[15] Sola R., Veronesi P., Giovanardi R., Forti A., Parigi G. (2017). Effect of heat treatment before cryogenic cooling on the proprieties of AISI M2 steel. Metall. Ital..

[16] Sola R., Giovanardi R., Parigi G., Veronesi P. (2017). A Novel Methods for Fracture Toughness Evaluation of Tool Steels with Post-Tempering Cryogenic Treatment. Metals.

[17] Shao H., Shan D., Bai L., Zhang G., Wang K., Zhao Y. (2018). Joule heating-induced microstructure evolution and residual stress in Ti-6Al-4V for U-shaped screw. Vacuum.

[18] Conghui Z., Shanshan Z., Yaomian W., Wei S. (2017). Effect of annealing on microstructure and residual stress of commercially pure zirconium with surface mechanical attrition treatment. Heat Treat. Met..

[19] Yang L., Li X.Y., Lu K. (2017). Making materials plain: Concept, principle and applications. Acta Metall. Sin..

[20] Wawszczak R., Baczmański A., Marciszko M., Wróbel M., Czeppe T., Sztwiertnia K., Braham C., Berent K. (2016). Evolution of microstructure and residual stress during annealing of austenitic and ferritic steels. Mater. Charact..

[21] Marzbanrad B., Jahed H., Toyserkani E. (2018). On the evolution of substrate’s residual stress during cold spray process:A parametric study. Mater. Des..

[22] Hsiao S.N., Chen L.H., Liu S.H., Tsai J.L., Lee H.Y. (2016). Evolution of microstructure, residual stress, and texture in FePt films during rapid thermal annealing. J. Alloy. Compd..

[23] Zhao J., Deng Y., Wei H., Zheng X., Yu Z., Shao Y., Shield J.E., Huang J. (2017). Strained hybrid perovskite thin films and their impact on the intrinsic stability of perovskite solar cells. Sci. Adv..

[24] Nakano S., Gao B., Kakimoto K. (2017). Numerical analysis of dislocation density and residual stress in a GaN single crystal during the cooling process. J. Cryst. Growth.

[25] Meng X.K., Zhou J.Z., Huang S., Su C., Sheng J. (2017). Properties of a Laser Shock Wave in Al-Cu Alloy under Elevated Temperatures: A Molecular Dynamics Simulation Study. Materials.

[26] Wang Y., Shi J., Ji C. (2014). A numerical study of residual stress induced in machined silicon surfaces by molecular dynamics simulation. Appl. Phys. A.

[27] Li P., Yang Y., Zhang W., Luo X., Jin N., Liu G. (2016). Structural evolution of TiAl during rapid solidification processing revealed by molecular dynamics simulations. RSC Adv..

[28] Wu H.N., Xu D.S., Wang H., Yang R. (2016). Molecular Dynamics Simulation of Tensile Deformation and Fracture of γ-TiAl with and without Surface Defects. J. Mater. Sci. Technol..

[29] Ruicheng F., Hui C., Haiyan L., Zhiyuan R., Changfeng Y. (2018). Effects of Vacancy Concentration and Temperature on Mechanical Properties of Single-Crystal γ-TiAl Based on Molecular Dynamics Simulation. High Temp. Mater. Process..

[30] Zhang B., Zhang X., Li C., Zhou K. (2012). Molecular Dynamics Simulation on Phase Transformation of Ti-Al Alloy with Low Al Content. Rare Met. Mater. Eng..

[31] Feng R.C., Lu J.T., Li H.Y., Cao H., Rui Z.Y. (2017). Effect of the Microcrack Inclination Angle on Crack Propagation Behavior of TiAl Alloy. Strength Mater..

[32] Zou J., Fu C.L., Yoo M.H. (1995). Phase stability of intermetallics in the Al Ti system: A first-principles total-energy investigation. Intermetallics.

[33] Yamaguchi M., Inui H., Ito K. (2000). High-temperature structural intermetallics. Acta Mater..

[34] Uezaki K., Shimizu J., Zhou L. (2014). Development of metal cutting process accompanied by a localized compressive hydrostatic stress field formation: Examination by molecular dynamics simulation. Precis. Eng..

[35] Tuckerman M. (2010). Statistical Mechanics: Theory and Molecular Simulation.

[36] Tao H.J., Sun S.P., Zhang C.C., Chen T., Luo W., Jiang Y. (2014). First principles of point defect concentrations in L1_0_−TiAl intermetallic composite. Chin. J. Nonferrous Met..

[37] Wang W.J., Wang T.M., Zhang H.T. (2001). Computer Simulation of Crystal defects in TiAl Alloys. J. Lanzhou Univ. (Nat. Sci.).

[38] Hammer R., Todt J., Keckes J., Defregger S. (2017). High resolution residual stress gradient characterization in W/TiN-stack on Si(100): Correlating in-plane stress and grain size distributions in W sublayer. Mater. Des..

[39] Hansen N. (2004). Hall–Petch relation and boundary strengthening. Scr. Mater..

[40] Park J.K., Jung J.Y., Chun C.H., Her S.M. (1996). Hall-Petch relation in lwo-phase TiAl alloys. Mater. Sci. Eng. A.

[41] Wei Y.J. (2014). Investigation of mechanical behavior of interfaces in nanostructured metals. Acta Metall. Sin..

[42] Shan Z., Stach E.A., Wiezorek J.M.K., Knapp J.A., Follstaedt D.M., Mao S.X. (2004). Grain Boundary-Mediated Plasticity in Nanocrystalline Nickel. Science.

